# Anti-high mobility group box protein 1 monoclonal antibody downregulating P-glycoprotein as novel epilepsy therapeutics

**DOI:** 10.1186/s41983-022-00557-8

**Published:** 2022-10-22

**Authors:** Bryan Gervais de Liyis, Sevinna Geshie Tandy, Joana Fourta Endira, Komang Andjani Putri, Desak Ketut Indrasari Utami

**Affiliations:** 1 Faculty of Medicine, Udayana University, Bali, Indonesia; 2 Department of Neurology, Faculty of Medicine, Udayana University, Bali, Indonesia

**Keywords:** Epilepsy, HMGB1, Monoclonal antibody, Therapy

## Abstract

Epilepsy, a neurological illness, is characterized by recurrent uncontrolled seizures. There are many treatments of options that can be used as the therapy of epilepsy. However, anti-seizure medications as the primary treatment choice for epilepsy show many possible adverse effects and even pharmacoresistance to the therapy. High Mobility Group Box 1 (HMGB1) as an initiator and amplifier of the neuroinflammation is responsible for the onset and progression of epilepsy by overexpressing P-glycoprotein on the blood brain barrier. HMGB1 proteins then activate TLR4 in neurons and astrocytes, in which proinflammatory cytokines are produced. Anti-HMGB1 mAb works by blocking the HMGB1, reducing inflammatory activity in the brain that may affect epileptogenesis. Through the process, anti-HMGB1 mAb reduces the TLR4 activity and other receptors that may involve in promote signal of epilepsy such as RAGE. Several studies have shown that anti-HMGB1 has the potential to inhibit the increase in serum HMGB1 in plasma and brain tissue. Further research is needed to identify the mechanism of the inhibiting of overexpression of P-glycoprotein through anti-HMGB1 mAb.

## Introduction

Excessive and hypersynchronous firing of neurons in the brain causes a seizure, which is a paroxysmal change of neurologic function [1]. The term "epileptic seizure" is used to differentiate a seizure produced by abnormal neuronal firing from a psychogenic seizure [2]. Recurring spontaneous uncontrolled seizures are referred to as epilepsy [3]. In 2015, epilepsy contributed around 125,000 fatalities worldwide and around 23 million prevalent cases [4]. In high-income nations, the condition has a lifetime prevalence of 2.3 to 15.9 per 1000 people, while in low-income countries it has a prevalence of 3.6 to 15.4 per 1000 people [5]. Epilepsy is more common in the first year of life and over 85 years with yearly estimates of 86 per 100,000 people and 180 per 100,000 people, respectively [6]. An estimated 75% of epilepsy cases begin in youth, owing to the increased sensitivity of seizures in developing brains [7]. Moreover, the most prevalent precipitating factors include fever, traumatic brain injury, cerebrovascular illness, substance withdrawal, inflammation, and hormonal disturbances [8]. A number of studies have suggested the role of P-glycoprotein (P-gp) in pathogenesis of epilepsy. P-gp is found to be responsible in limiting wide ranges of drugs in epilepsy therapy [9].

Although there are a variety of other medication strategies for epilepsy, such as vagus nerve stimulation [10], surgery [11], and a ketogenic diet [12], anti-seizure medications (ASMs) are typically the first option [13]. The majority of data for the efficacy and safety of ASMs comes from subjects under 65 years old, and there are few randomized controlled studies in older individuals [14]. Pharmacokinetics shift, comorbidities, physiological alterations, polypharmacy and accompanying impairment of capability may complicate the administration and effectivity of ASMs in older age groups [15, 16]. Additionally, drug interactions and possible adverse effects, such as nausea, sleepiness, mental retardation, rashes, liver toxicity and metabolic changes should be properly examined while using ASMs [17]. Furthermore, more than half of epilepsy patients using ASMs have been reported to acquire bone abnormalities [17, 18], and numerous studies have found a connection between extensive ASM usage with rickets, osteomalacia [19] and increased risks of fracture [20].

HMGB1 (High Mobility Group Box 1) is a non-histomic, chromosome-linked small protein with cytokinin function that has nuclear, cytosolic, and extracellular activities [21]. Being the dominant nonhistone nucleoprotein in the HMGB gene family, HMGB1 shuttles between nucleus and cytoplasm serving as both a nonhistone nucleoprotein as well as an inflammatory cytokine [22]. Through TLR4/RAGE/NF-kB pathway activation in brain microvascular endothelium, HMGB1 leads to P-gp overexpression in epileptic brain regions [23, 24]. Due to its overexpression during seizures, P-gp has been intensively researched for developing multidrug resistance in epilepsy [25]. Thus, through the hyperexcitability of the HMGB1/RAGE/TLR4/NF-kB signaling pathway, HMGB1 has a pro-epileptic impact that induces temporal lobe epilepsy development [26–29]. This finding is also congruent with research conducted by Paundel et.al that proposed inhibiting the HMGB1/RAGE/TLR4/NF-kB pathway might be a potential treatment approach against HMGB1-mediated epileptic disorders [30]. Inhibiting the signaling pathway downregulate P-gp overexpression and it might be studied as a potential therapeutic approach [24, 31]. Fortunately, recent studies show that anti-HMGB1 monoclonal antibodies (mAb) treatment might be effective in treating a wide range of central nervous system (CNS) and peripheral nervous system (PNS) disorders such as epilepsy [32, 33]. In acute stage of status epilepticus, intravenous therapy with neutralizing anti-HMGB1 monoclonal antibody exhibited neuroprotective effect on neuronal death, inhibition of HMGB1 release, blood brain barrier (BBB) protection and anti-inflammatory actions [34].

The goal of this review is to evaluate the prospects of anti-HMGB1 monoclonal antibodies in downregulating P-glycoprotein as a therapeutic approach in epilepsy management.

## The pathophysiology of epilepsy

The brain is responsible to control the activity of neurons, nerve cells which specially designed to channel electrical impulses around the central and peripheral nervous systems [35]. A seizure developed when an abnormal synchronous firing of neurons in some parts of the brain occurs [36]. A patient with seizure experienced certain distraction of the normal balance involving excitation and inhibition which happened in the brain [7]. Normal membrane conductance, inhibitory synaptic current breakdown and excessive excitability spread locally resulting in a focal seizure or widely resulting in generalized seizure [37].

Many acquired brain pathologies, such as tumors, stroke, infection, traumatic brain injury, and mutation of a single gene contribute to the presentation of epilepsy [38]. Some evidence shows that neuroinflammation contributes to the onset and progression of epilepsy alongside HMGB1 as an initiator and amplifier of the process [39]. In the inflammatory process of epileptic brain, the activity and level of P-gp escalate on the BBB with overexpression as the result [23]. The inhibition and induction of P-gp is known as essential underlying components of drug interactions in humans [40]. There are different theories interpreting the nature of drug resistance with the pharmacokinetic hypothesis and the transport hypothesis of the drug, displaying the possible mechanism of the resistance in ASMs by the overexpression of P-gp in the BBB [41]. The P-gp distribution is arranged to narrow drug entry to the brain or letting efflux of drugs from brain to blood, which reveals that P-gp plays an essential part in protecting the brain against xenobiotics [42]. Thus, the crucial efflux protein of the BBB is P-gp that repeatedly transports enormous amounts of lipophilic drugs, as well as ASMs, out of the BBB membrane and back toward the bloodstream, ended up in pharmacoresistance to therapeutic medications intended to target the brain [43]. Individuals who fail to respond or only partially respond to ASMs will continue to experience paralyzing seizures that lead to psychiatric, neuropsychological, and social impairments by such means decreasing the quality of life and increasing morbidity and mortality [44].

## HMGB1 in expressing P-glycoprotein

**Fig. 1:**
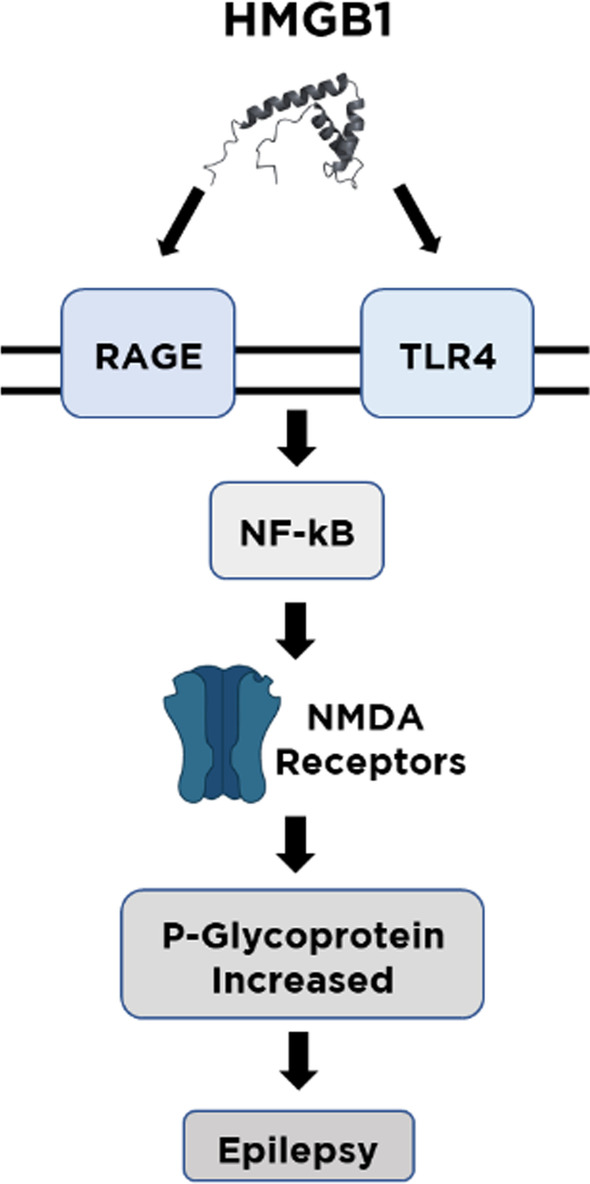
HMGB1/RAGE/TLR4 pathway signaling. RAGE; receptor for advanced glycation end products, TLR4; toll like receptor 4, NF-kB; nuclear factor kappa B, NMDA; *N*-methyl-D-aspartic acid

Seizures begin with a strong stimulation of vulnerable epileptic neurons, which leads to gradual induction of linked neurons [45]. Uncontrolled impulses finally impact a portion of the brain and cause clinical symptoms [46]. In a patients epileptic brain study, researchers discovered higher levels of HMGB1, TLR4, RAGE, NF-kB and nitric oxide synthase, as well as higher amounts of IL-1, IL-6, TNF- α, TGF- β, and IL-10 [47]. The ligand–receptor effects of HMGB1-RAGE/TLR4 in the generation of seizures have been clearly established [48]. A rise in extracellular HMGB1 paralleled seizure onset, confirming HMGB1 as a significant contributor to the beginning of the hyperexcitability [49]. The activation of neuron TLR4 by HMGB1 is a critical process in seizure onset [27]. Hence, HMGB1/RAGE/TLR4 pathway may initiate the development and persistence of seizures in people (Fig. 1) and can be addressed to achieve anti-seizure results in drug-resistant epilepsies [30].

RAGE aids TLR4 trafficking to the cell surface, while TLR4 controls RAGE translation and translocation to cell surface [50]. The TLR4 receptor complex stimulates the recruitment of different adaptor proteins and activates multiple signaling pathways, ultimately activating NF-kB [51]. HMGB1 promotes nuclear translocation of nuclear factor-B, resulting in the production of proinflammatory cytokines such as TNF-α, IL-6 and IL-1β [52]. Through the N-methyl-D-aspartate (NMDA)/cyclooxygenase-2 (COX-2) pathway by NF-kB, extracellular glutamate enhanced the amount of P-gp expression [23]. TNF-α, an inflammatory cytokine, has been shown to promote P-gp activity in the BBB [53].

## Anti-HMGB1 monoclonal antibody in epilepsy

In a clinical study involving adult zebrafishes, anti-HMGB1 mAb reduces seizures, improves associated cognitive deficits, and reduces seizure-induced elevation of proinflammatory cytokines, potentially protecting from future seizures through neuroinflammatory network regulation [54]. Intravenous administration of anti-HMGB1 mAb protects rats against BBB rupture caused by infarction and hemorrhage, and this protective activity is coupled with the reduction of production of inflammatory mediators markers and glial cell stimulation [55]. In modeled animals, the anti-HMGB1 monoclonal antibody (mAb) has also been proven to be useful for the treatment of a variety of CNS illnesses, notably hemorrhage, brain trauma, Parkinson's disease and Alzheimer's disease [33]. Although still in the phase of animal model clinical trials, researchers support the potential use of the substance in humans to downregulate the elevated HMGB1 [39].

**Fig. 2:**
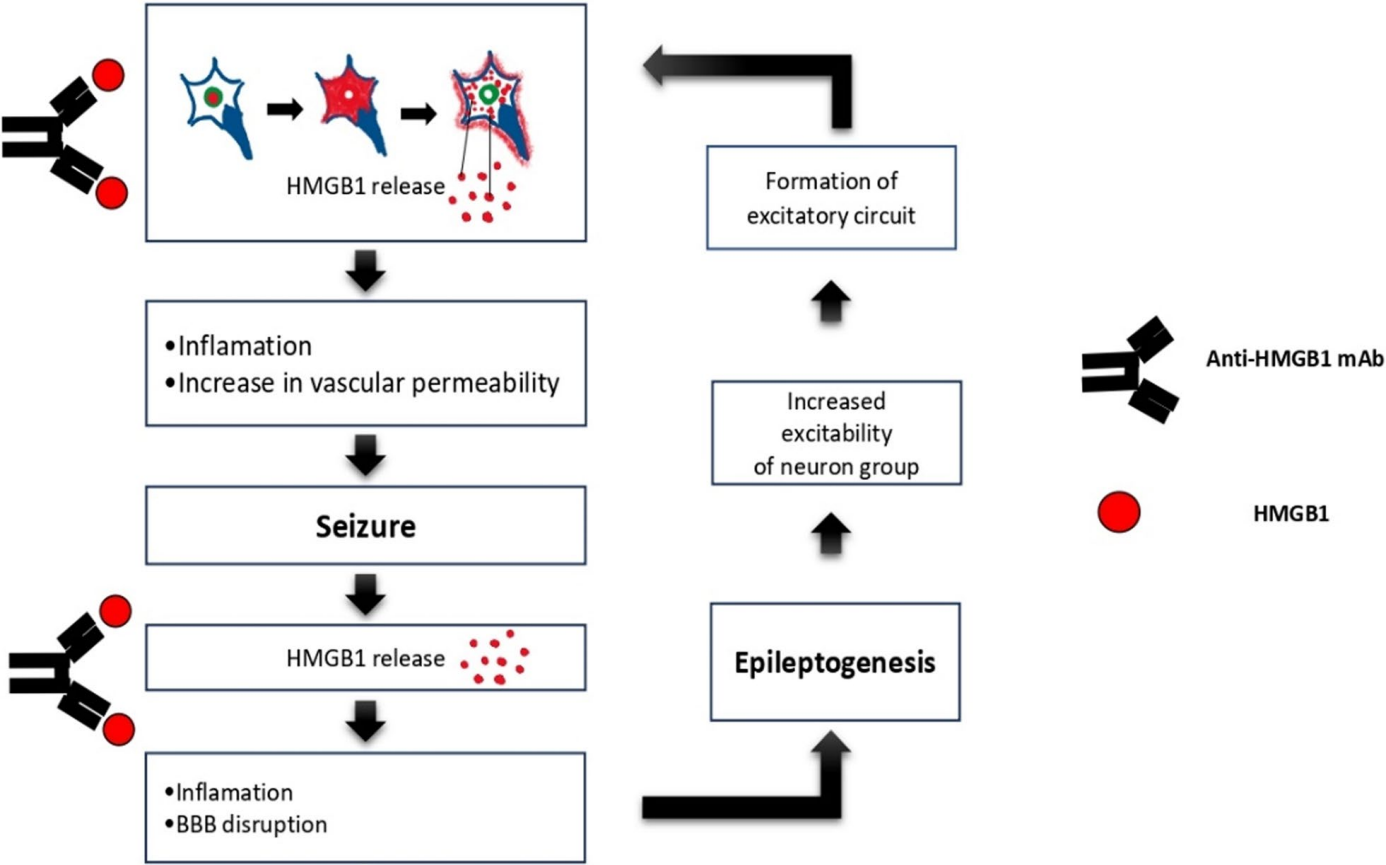
Anti-HMGB1 mAb can inhibit status epilepticus and epileptogenesis. HMGB1; High Mobility Group Box 1, BBB; blood brain barrier

HMGB1 will continue to increase during seizures and may holds a role in upregulating P-gp [33]. Some theories point out involvement of P-gp overexpression in status epilepticus [24]. Several experiments showed ASM being pumped by P-gp, resulting in low concentration of ASM in brain tissue [33, 54]. Nishibori et al. reported that the epileptiform activity in the brain slices was suppressed by superfusion of anti-HMGB1 mAb on the surface of brain slices taken from patients who had repeated seizures. Anti-HMGB1 mAb can be useful in the treatment of various types of inflammatory diseases that are specific to BBB disruptions in animal models [33]. Disruption of BBB may be caused by the participation of extracellular HMGB1 [56]. One of the current therapeutic strategies for epilepsy is to focus on molecular targeting of HMGB1 with specific antibodies or antagonists that have the potential to minimize the occurrence of epileptic seizures characterized by HMGB1 expression [39]. HMGB1, IL-1β, and S-100B can be molecular markers in the incidence of epilepsy characterized by changes in serum concentrations so that they have clinical significance in the epileptic process and can predict outcomes [57]. Anti-HMGB1 mAb has good drug potency with high therapeutic index and sufficient specificity to be used as therapy. Administration of anti-HMGB1 mAb and diazepam was shown to reduce the number of generalized seizures [58]. Anti-HMGB1 can help to regulate the occurrence of epilepsy and the process of epileptogenesis (Fig. 2) [26, 30, 33]. Epileptogenesis is a process when the brain under normal conditions undergoes functional changes in the form of abnormal electrical activity, resulting in chronic seizures. In general, epileptogenesis consists of three stages, namely acute events or precipitating events, latent or clinically silent periods, and stages of chronic epilepsy or spontaneous seizures [59–61]. Trigger factors can be in the form of oxidation, inflammation, neurogenesis, apoptosis, and synaptic plasticity. These factors can lead to functional and structural changes in nerve cells. These changes result in abnormal hyperexcitability and spontaneous seizures [62].

Convulsant activation also occurs due to IL-1β and HMGB1 which result in neuronal adjustment, leading to a decrease in the threshold for focal ictal events. [63]. There will be activation of astrocytes and microglia through an increase in the number and shape of specific cells that occur in the cerebral cortex and hippocampus during acute epilepsy [34]. Activation of glial cells initiates epileptogenesis after status epilepticus. IP3 R2 which mediates Ca2 + signaling in reactive astrocytes will be proconvulsive in the epileptic brain and contribute to epileptogenesis. Activation of microglia is the initial process of epileptogenesis which is indicated by the increase of proinflammatory cytokines of microglia before the activation of astrocytic [64].

Anti-HMGB1 mAb can inhibit the activation of these cells systemically. Anti-HMGB1 mAb can reduce the number of cells undergoing apoptosis after the occurrence of acute status epilepticus [34]. HMGB1 translocation of neurons and astrocytes can be inhibited by anti-HMGB1 which can decrease the duration of tonic–clonic seizures by increasing the threshold [65]. HMGB1 is a molecule that plays an important role in increasing BBB permeability in the acute phase of epilepsy. HMGB1 released into the CNS and peripheral blood stream after pilocarpine injection plays a role in inducing damage to the BBB. Anti-HMGB1 can significantly inhibit the permeability of the BBB [34]. In a study involving epilepsy by inducing upregulation of IL-1β and TNF-α mRNA was shown to be blocked by anti-HMGB1 through a potential anti-inflammatory mechanism that could inhibit microglia activation and proinflammatory cytokine synthesis [66]. Expression of proinflammatory cytokines also occurs as a result of an excess of HMGB1 and will subsequently have an impact on the continuous induction of HMGB1 release [67]. In addition to its anti-inflammatory effect, anti-HMGB1 mAb has a mechanism in inhibiting the increase of serum HMGB1 in brain tissue and plasma as well as interfering with the translocation of HMGB1 from the nucleus to astrocytes and neuronal cytoplasm, thus forming BBB stabilization [68]. Anti-HMGB1 also can interrupt TNF-α formation and expression by western blot analysis [69]. Complex cellular internalization of HMGB1 and HMGB1-LPS can be inhibited by anti-HMGB1 mAb, HMGB1 antagonist box A protein, nicotinic acetylcholine receptor subtype alpha 7 GTS-21 agonist, and acetylcholine [70]. Through TLR4/NF-κB and RAGE/NF-κB, HMGB1 may have an effect on increasing P-gp expression in seizure-induced rat brain endothelial cells [23]. Excessive activation of P-gp results in decreased focal (R)-[^11^C] uptake of verapamil in pharmacoresistance seen in pharmaco-resistant temporal lobe epilepsy in an in vivo study [25]. Inhibition of HMGB1 with anti-HMGB1 mAb has an important role in obtaining therapeutic benefit in the incidence of seizures and their epileptogenesis [26, 39, 57].

Many experiments have been done by using animal models to observe involvement of HMGB1 in status epilepticus [33]. Finding by Fu et al. in observing hippocampal neurons to see significant translocation of HMGB1 after the pilocarpine injection and systemic injection of anti-HMGB1 mAb that significantly inhibited HMGB1 translocation, thereby suppressing the status epilepticus. In this model, administration of anti-HMGB1 gives protection effects against neuronal apoptosis, avoiding the BBB disruption and resisting the inflammation effect by pilocarpine [34]. An experiment by Zhao et al. using 4 types of epileptic animal models points out the beneficial effects using systemic injection of anti-HMGB1 mAb as the treatment. These animal models include the kainite-induced epilepsy, chronic seizure-induced epilepsy, electroshock-induced epilepsy and picrotoxin-induced epilepsy [65].

## Pharmacokinetics and pharmacodynamics of anti-HMGB1 monoclonal antibody

Monoclonal antibody already being developed as clinical therapeutics nowadays [71]. Anti-HMGB1 impacts several central nervous system pathological conditions, such as Parkinson disease, traumatic brain injury, stroke, and spinal cord injury [33, 68, 72–74]. In dose-dependent manner, anti-HMGB1 monoclonal antibody (mAb) extended the generalized seizure’s latency, decreased tonic convulsion, and reduced seizure stage [65]. According to Okuma et al., in treatment of brain injury, the anti-HMGB1 mAb dosage is 1 mg/kg was administrated intravenously at 5 min and 6 h after injury [72]. Anti-HMGB1 mAb was diluted by 2 mg/ml phosphate-buffered saline [65]. Anti-HMGB1 mAb injected intravenously in rat experiments shows the anti-HMGB1 accumulation in ischemic site of the brain in PET imaging 16 h after injection. Small amount of anti-HMGB1 is also present in brain parenchyma. Anti-HMGB1 possible to spread into blood brain barrier and luminal side of vascular endothelial cell [33]. Anti-HMGB1 only targeting HMGB1 (prevent translocation from nuclei) may be safer treatment to treat epilepsy [65]. Anti-HMGB1 mAb binds solidly with HMGB1. Thus, anti-HMGB1 estimated have long time elimination in the brain [75]. In general, monoclonal antibody is mostly eliminated by lysosomal degradation to amino acids. The molecule of monoclonal antibody is too large to eliminate by the kidney [76].

Major aim of epilepsy therapy is not only about reduction of seizure frequency, but also how to prevent epileptogenesis, the process of the brain develops epilepsy [77]. Anti-HMGB1 mAb blocking the HMGB1 resulting in reduced of inflammatory activity in the brain that may affect epileptogenesis [65]. Intraperitoneal injection of anti-HMGB1 mAb in previous study, effectively reduced the presence of HMGB1 in the nuclei cell of central nervous system. Anti-HMGB1 monoclonal antibody neutralized extracellular HMGB1 in the CNS resulted in persistent nuclear HMGB1 staining and alleviated inflammation [78]. Previous report found, anti-HMGB1 mAb effectively reduced brain ischemia by efficient clearance of HMGB1 and inhibiting edema, ameliorates spontaneous arthritis model, prevent joint destruction, and repair cardiac pathological changes in experimental autoimmune myocarditis by suppress Th-17 cell [75, 79, 80]. Anti-HMGB1 mAb have immunohistochemical and neurochemical benefit reducing HMGB1 level in the plasma and brain evaluating by ELISA in day 1,4,7, 14 after injection [68]. HMGB1 translocation from the nuclei to the cytosol is inhibited by anti-HMGB1 mAb. Blocking of HMGB1 by regulatory axis using anti-HMGB1 mAb, reduced the TLR4 and others receptor that may involve in promote signal of epilepsy such as RAGE [27]. HMGB1 is strictly involved in the inflammatory response related to epilepsy [81].

A study reported the therapeutic effect of anti-HMGB1 mAb on epilepsy start from 1 h and last to 24 h, indicating that there’s a potential clinical epilepsy treatment. In addition, the potential of anti-HMGB1 mAb on human epileptic brain slices has not been eliminated, suggesting that anti-HMGB1 mAb binds strongly to HMGB1 with potential long-term anti-epileptic activity [39, 65]. Anti-HMGB1 mAb only targets the translocated and activated HMGB1. Moreover, it is reported on previous study that anti-HMGB1 in a high dose (25 mg/kg in mice) did not affect body growth rate, basic physical functions and thermoregulation [65]. Although anti-HMGB1 mAb showed lots of potential for specificity therapy with wider therapeutic window, this strategy targeting HMGB1 is restricted by problems like the probability in the tertiary structure by conformational switches of the Ab-recognized zone [82].

The use of anti-hmgb 1 mAb as a modality of epilepsy therapy may focus on the cost component, which depends on the antibody protein used and the encapsulation of the antibody therapy so that it can enter the human body and reach its targets. From the antibodies used, as with mAb therapy in COVID-19, the costs incurred in mAb therapy in patients with epilepsy may be more expensive than previously available conventional modalities [83]. The potential for monoclonal antibody therapy in the future will be seen from the prevalence of disease incidence from the indications of therapy given. mAb therapy in oncological conditions with a smaller market certainly has a higher cost compared to immunological conditions with a larger market [84]. The second is from the formulation of therapeutic protein encapsulation. The therapeutic potential possessed by anti-hmgb1 mAb in the future has the opportunity for further development in terms of the formation of long-acting protein formulations so as to maintain protein stability and can provide appropriate doses for a longer duration of time. This needs to be developed in the future because of the limitations possessed by a mAb, namely from its short pharmacokinetic properties, protein stability, protein aggregation, and denaturation that may occur due to the storage and transportation process of the protein which will ultimately have an impact on its clinical use. The common way to stabilize protein-based drugs is through improving the molecular structure with protein engineering and administering stabilizers. Several formulations are being developed, such as through a controlled release system (hydrogel, micelles, liposomes, nanoparticles) [85, 86]. Polyester-based nanoparticles as one of the strategies developed in mAb encapsulation are said to have the potential to increase antibody stability, prolong the duration of the therapeutic effect, and recognize selective cellular targets [87]. Therefore, anti-HMGB1 mAb that significantly blockade the HMGB1 is potential treatment for epilepsy and assessing the efficacies with several experiments of epilepsy models needed to be the objectives for future research.

## Conclusion

Anti-HMGB1 mAb may have the potential to downregulate P-glycoprotein expression which is overexpressed due to an increase in HMGB1 in epilepsy. Several previous studies have shown that anti-HMGB1 can potentially inhibit the increase in serum HMGB1 in plasma as well as brain tissue and can interfere with the translocation of HMGB1 from the nucleus to astrocytes and neuron cytoplasm. Inhibition of HMGB1 by anti-HMGB1 has a significant clinical impact. Therefore, inhibition of the overexpression of P-glycoprotein via anti-HMGB1 mAb may be able to become a therapeutic target for epilepsy. The mechanism that occurs from the inhibition of overexpression of P-glycoprotein through anti-HMGB1 mAb needs further research. Anti-HMGB1 mAb may contribute to the development of anti-seizure medications in the future through its mechanism of action against P-glycoprotein.

## Data Availability

Not applicable.
